# A Bayesian model with seasonal effects for predicting accrual in clinical trials: Application to HOBIT and BOOST-3 trials for severe traumatic brain injury

**DOI:** 10.1016/j.conctc.2025.101586

**Published:** 2025-12-05

**Authors:** Mohammod Mahmudur Rahman, Md Saiful Islam Saif, Jonathan Beall, Renee’ L. Martin, Gaylan L. Rockswold, William G. Barsan, Frederick K. Korley, Robert Silbergleit, Valerie Stevenson, Byron Gajewski

**Affiliations:** a Department of Biostatistics & Data Science, University of Kansas Medical Center, Kansas City, KS, USA; b Department of Public Health Sciences, Medical University of South Carolina, Charleston, SC, USA; c Department of Neurosurgery, University of Minnesota, Hennepin County Medical Center, Minneapolis, MN, USA; d Department of Emergency Medicine, University of Michigan, Ann Arbor, MI, USA

**Keywords:** Accrual prediction, Seasonal trend, Hyperbaric oxygen brain injury treatment trial, Brain oxygen optimization in severe TBI Phase-3 trial

## Abstract

**Background:**

Accurate accrual prediction is essential for initial planning and ongoing monitoring of clinical trials. Slow accrual can compromise statistical power, increase costs, or lead to premature trial termination. Traditional Bayesian approaches typically assume constant accrual rates and often fail to capture real-world seasonal fluctuations, which can reduce predictive accuracy.

**Methods:**

We developed a Bayesian seasonal accrual model that extends the traditional homogeneous model by incorporating quarter-specific priors to account for seasonal variation. The model combines prior knowledge with observed data up to the monitoring point to obtain accrual predictions using the Bayesian posterior predictive distribution. We applied this approach to quarterly accrual data from two ongoing trials: the Hyperbaric Oxygen Brain Injury Treatment (HOBIT) trial and the Brain Oxygen Optimization in Severe TBI Phase-3 (BOOST-3) trial. Along with the Deviance Information Criterion, model performance was evaluated using RMSE, bias, and standard deviation, calculated from internal predictions of total accruals within observed seasonal quarters. Posterior predictive distributions of accrual after 36 and 30 quarters were also generated.

**Results:**

Both trials exhibited seasonal trends, with the highest accrual rates in summer. The seasonal model yielded lower DIC in both trials. In HOBIT, internal prediction accuracy did not improve, likely due to uniformly low accrual rates. In contrast, the seasonal model outperformed the homogeneous model in BOOST-3 trial, yielding substantially lower RMSE, bias, and SD.

**Conclusion:**

Incorporating seasonal effects into accrual modeling can enhance prediction accuracy, particularly in larger trials with high enrollment, and supports more accurate trial forecasting and resource allocation.

## 1 Introduction

Accrual in Clinical trials poses a major challenge due to the substantial costs associated with achieving the requisite sample sizes [[1], [2], [3]]. Previous studies show that 86 % of trials do not finish on time and fail to meet their accrual targets [2,4], and 19 % of eligible trials either terminated for failed accrual or completed with less than 85 % of the expected enrolment [5]. The Swiss National Science Foundation (SNSF) reported that 25 % of SNSF-supported trials were prematurely discontinued due to slow accrual [6]. A reinvestigation of the recruitment in trials funded by two UK funding agencies also revealed that 45 % of trials failed to achieve their target sample size [7].

Predicting participant accrual is essential for the initial planning and ongoing monitoring of clinical trials as well as for ensuring adequate statistical power and effectively managing the resources needed to sustain the trial. Various modeling approaches have been proposed in the literature to predict patient accrual in clinical trials. Comfort [8] proposed a conditional deterministic model termed a second-order recruitment model (SORM). It used a piecewise approach where the recruitment rate depends on the number of centers open during the initial phase. Senn [9] proposed an accrual model based on a homogeneous Poisson process, assuming all centers have a constant enrollment rate. This framework utilized a gamma distribution to find the accrual time for a desired sample size. Lee [10] developed an accrual model based on setting interim goals using the observed enrollment rate at stage k to predict the rate for stage k+1. Zhang and Long [11] employed B-splines to model variations in accrual rates over time that can capture dynamic changes in recruitment. Tang et al. [12] proposed a discrete-time Poisson process model with three phases that assumes an initiation phase with an increasing accrual rate, a second phase where the accrual rate is constant, and a final surge that follows the announcement of the closing date. Lai et al. [13] utilized Brownian motion to model cumulative enrollment as a linear function of time. Anisimov and Fedorov [14] proposed a recruitment model within an empirical Bayesian setting where patients’ arrival at different centers follow Poisson processes, and the arrival rates are modeled as samples drawn from a gamma distribution. Gajewski et al. [15] proposed a Bayesian framework that assumes the waiting time between consecutive patient arrivals follows an exponential distribution and used an inverse gamma distribution as the prior for the arrival rate. Later, Jiang et al. [16] extended this model by introducing various adaptive priors based on clinical data. Liu [17] proposed a Bayesian hierarchical model that accounts for varying center activation times to describe the patient accrual across multiple centers.

Evidence also shows that accrual is influenced by seasonal factors. A previous study based on a large database included all clinical studies launched by the AIDS Clinical Trials Group (ACTG) between October 1986 and November 1999, and indicated that patient enrollment in trials differed across different months of the year, with peaks observed in spring and late fall [18]. By utilizing an autoregressive time series model, it predicted enrollment for future quarters. Lee's method offers a flexible framework that allows for incorporating seasonal adjustments depending on the type of diseases [10]. Lan and Tang et al. [19] developed a method for predicting trial recruitment that incorporates center initiation times, between-center heterogeneity in enrollment capacity, and declining trends in enrollment within initiated centers. Villasante-Tezanos and Kuo et al. [20] proposed a weighted resampling-based nonparametric approach to predict accrual, which incorporates seasonal factors by assigning weights and acknowledges that seasonal variations significantly influence patient recruitment patterns. Although several approaches have addressed seasonal effects in accrual prediction, to our knowledge, no research has incorporated this effect into Bayesian modeling for accrual prediction. Ignoring seasonality can result in inaccurate accrual predictions, particularly in emergency medicine trials conducted across multiple years, where holidays and weather conditions can strongly influence accrual.

In this study, we extend the homogeneous accrual model proposed by Gajewski et al. [15] by incorporating seasonal variations through quarter-specific priors and posterior predictive distributions. We introduce seasonality using calendar quarters, since it aligns with the four meteorological seasons (winter, spring, summer, and fall), and emergency department and trauma center admissions are well known to vary by season. Several previous studies have examined the effects of weather and season on hospital admissions for traumatic injuries and found that the incidence of trauma-related admissions largely depends on the season, with significantly higher rates in summer than in other seasons [[21], [22], [23]]. We applied our approach to two ongoing severe traumatic brain injury trials: the Hyperbaric Oxygen Brain Injury Treatment (HOBIT) trial and the Brain Oxygen Optimization in Severe TBI Phase-3 (BOOST-3) trial. We compared our proposed seasonal model with the homogeneous model by performing internal predictions of total accrual using a varying number of observed quarters, and utilized Root Mean Squared Error (RMSE), bias, and standard deviation (SD) as the evaluation measures. Additionally, posterior predictive distributions of total accrual after 36 and 30 quarters were generated for HOBIT and BOOST-3 trials, respectively, to assess the long-term effects of seasonality.

## 2 Methods

### 2.1 Homogeneous model

The homogeneous accrual rate model proposed by Gajewski et al. [15] assumes the patient accrual rate remains constant across all quarters with no seasonal variation. Hereby, we referred to this model as “Homogeneous Model.” Let J denote the total number of observed quarters of a trial, with j=1,2,…,J. Let $m_{j}$ represent the number of patients accrued during the j-th quarter, and $t_{j\;}$ denote the duration of j-th quarter expressed as a fraction of a full quarter.

Under the homogeneous Poisson assumptions, the accrual count can be defined as:

$$m_{j}∼\text{Poisson}\left(\mu_{j}\right)\text{,}$$

with $\mu_{j}=\lambda \times t_{j\;}$ where λ is the constant accrual rate. A prior distribution of λ can be specified as Gamma(α,β) with density $\pi \left(\lambda \right)=\frac{\beta^{\alpha }}{\Gamma \left(\alpha \right)}\lambda^{\alpha -1}e^{-\beta \lambda },\lambda >0,\alpha >0,\beta >0$.

By combining the Poisson likelihood with the gamma prior, the posterior distribution for λ becomes as follows:

$$p\left(\lambda |m_{j},t_{j}\right)\propto \prod_{j=1}^{J}\left[\frac{{\left(\lambda \cdot t_{j}\right)}^{m_{j}}e^{-\lambda \cdot t_{j}}}{m_{j}!}\right]\cdot \frac{\lambda^{\alpha -1}e^{-\beta \lambda }}{\Gamma \left(\alpha \right)}\text{.}$$

To derive the posterior, we simplified the above equation:

$$\lambda \;|\;m_{j},t_{j}\propto \lambda^{\sum_{j}m_{j}+\alpha -1}\;\exp\left(-\lambda \left(\sum_{j}t_{j}+\beta \right)\right)\text{.}$$

It is the kernel of the Gamma distribution, and therefore, the posterior distribution is

$$\lambda \;|\;m_{j},t_{j}∼\text{Gamma}\left(\alpha +\sum_{j=1}^{J}m_{j},\beta +\sum_{j=1}^{J}t_{j}\right)\text{.}$$

Let q denote the number of quarters for which we aim to obtain the posterior predictive distribution of total accrual. For each new quarter up to the q-th quarter, we generated predictive accruals using draws from the posterior distribution of λ. For every new quarter *j* with duration $t_{j}$, we first simulated a draw from the posterior distribution of λ, and used that sampled value as the parameter in the predictive distribution. Therefore, the posterior predictive distribution for the *j*-th new quarter becomes

$$m_{pred,j}∼\text{Poisson}\left(\lambda^{*}.t_{j}\right)\text{,}$$

where $\lambda^{*}$ is a random draw from the posterior distribution of λ. The predicted accruals for all new quarters were then summed with the observed accruals from completed quarters. This process was repeated 10,000 times to obtain the posterior predictive distribution of total accrual.

### 2.2 Seasonal model

We propose an extension of the homogeneous accrual rate model by incorporating seasonal variability into the accrual process and refer to it as the “Seasonal Model.” Suppose each quarter *j* belongs to a season denoted by $S_{\left(j\right)}$, where $S_{\left(j\right)}\in \left\{1,2,3,4\right\}$ represents the seasons Summer, Fall, Winter, and Spring, respectively. The accrual count for quarter *j* is defined as:

$$m_{j}∼\text{Poisson}\left(\lambda_{S_{\left(j\right)}}\cdot t_{j}\right)\text{,}$$

where $\lambda_{S_{\left(j\right)}}$ is the accrual rate corresponding to the season assigned to quarter *j*. Let $J_{s}=\left\{j:S_{\left(j\right)}=s\right\}$ denote the set of quarters corresponding to a season s. We define $\lambda_{s}$ as the season-specific accrual rate, with each rate independently following a Gamma prior:

$$\lambda_{s}∼\text{Gamma}\left(\alpha ,\beta \right)\text{.}$$

The posterior distribution for $\lambda_{s}$, given the observed data from the quarters in season s is:

$$\lambda_{s}|{\left\{m_{j},t_{j}\right\}}_{{j\in J}_{s}}∼\text{Gamma}\left(\alpha +\sum_{j\in J_{s}}m_{j},\beta +\sum_{j\in J_{s}}t_{j}\right)\text{.}$$

Posterior predictive distribution of total accruals up to the q-th quarter was generated using season-specific posterior samples of $\lambda_{s}$. For each new quarter *j*, the predicted accrual was simulated as:

$$m_{pred,j}∼\text{Poisson}\left(\lambda_{S_{\left(j\right)}}^{*}\cdot t_{j}\right)\text{,}$$

where $\lambda_{S_{\left(j\right)}}^{*}$ is a simulated draw from the posterior distribution of $\lambda_{s}$. The predicted accruals for all new quarters were then summed with the observed accruals from completed quarters, and this process was repeated 10,000 times for each season.

## 3 Clinical trial data description

### 3.1 The Hyperbaric Oxygen Brain Injury Treatment (HOBIT) trial

The Hyperbaric Oxygen Brain Injury Treatment (HOBIT) study [24,25] is a multicenter randomized clinical trial designed to evaluate the efficacy of different hyperbaric oxygen therapy regimens for patients with severe traumatic brain injury (TBI). The goal of the trial is to identify the optimal oxygen treatment strategy, defined as hyperbaric oxygen with or without normobaric oxygen at varying pressure levels, that maximizes the likelihood of favorable neurological outcomes when compared to standard care. HOBIT trial started on June 25, 2018, and it aimed to enroll 200 participants across 14 centers over a 48-month period (16 quarters). As of December 16, 2024, it enrolled 139 participants, and the trial is still ongoing.

### 3.2 The Brain Oxygen Optimization in Severe TBI Phase-3 (BOOST-3) trial

The Brain Oxygen Optimization in Severe TBI Phase-3 (BOOST-3) study [26] is a multicenter, randomized, blinded, and adaptive Phase III trial focused on comparing the effectiveness of two standard treatment strategies for patients with severe TBI admitted to the intensive care unit (ICU). Specifically, the trial compares the effectiveness of two monitoring-based strategies: a conventional approach guided solely by an intracranial pressure (ICP) monitor, and an enhanced strategy guided by both ICP and brain tissue oxygenation (PbtO_2_) monitors. BOOST-3 trial started in August 2019 and was designed to enroll up to 1094 participants over a four-year period. However, by December 20, 2024, the trial had enrolled 725 participants, which is below the planned target, and the study remains ongoing.

### 3.3 Statistical analyses

Statistical analyses were conducted to fit both homogeneous and seasonal models for the HOBIT and BOOST-3 trials. The models estimated posterior distributions for the accrual rate and generated posterior predictive distributions of patient accruals for future quarters. Markov Chain Monte Carlo (MCMC) sampling was used to draw Bayesian inference. All analyses were implemented in R version 4.5.0 and OpenBUGS.

### 3.4 Season specifications

Accrual data for both trials were recorded every month, and we aggregated them into seasonal quarters (i.e., summer, fall, winter, and spring). Each seasonal quarter was defined as a three-month interval: summer (mid-June to mid-September), fall (mid-September to mid-December), winter (mid-December to mid-March), and spring (mid-March to mid-June). The HOBIT trial began in mid-June, which is the start of the summer season. For the BOOST-3 trial, although data collection began in mid-August, only one participant was enrolled during that partial quarter of summer. Due to the incompleteness of that seasonal quarter, we excluded that observation and defined fall as the starting point for patient accrual in BOOST-3.

### 3.5 Prior specifications

For the HOBIT trial, the total planned enrollment was 200 participants over a study duration of 48 months, which corresponds to 16 quarters. This implies an expected accrual rate of 12.5 participants per quarter. Using a confidence level of p=0.5, we defined the shape and scale parameters of the Gamma prior for the homogeneous model as: α=12.5×p=12.5×0.50=6.25 and β=1×p=0.5. For the seasonal model, we assigned the same gamma prior independently and identically to each $\lambda_{S_{\left(j\right)}}$, where $S_{\left(j\right)}\in \left\{1,2,3,4\right\}$. Thus, the prior distribution for the accrual rates in the homogeneous and seasonal models were defined as:

λ∼Gamma(α=6.25,β=0.5)

$$\lambda_{S_{\left(j\right)}}∼\text{Gamma}\left(\alpha =6.25,\beta =0.5\right)\text{.}$$

For the BOOST-3 trial, the planned enrollment was 1094 participants over the same duration of 48 months or 16 quarters, corresponding to an expected accrual rate of approximately 68.37 participants per quarter. Using the same prior specification strategy as in the HOBIT trial, we set the shape and scale parameters to: α=68.37×p=12.5×0.50=34.18 and β=1×p=0.5. Accordingly, we used the prior distribution for the accrual rate in both the homogeneous and seasonal models for the BOOST-3 trial as:

λ∼Gamma(α=34.18,β=0.5)

$$\lambda_{S_{\left(j\right)}}∼\text{Gamma}\left(\alpha =34.18,\beta =0.5\right)\text{.}$$

### 3.6 Model implementation

For the homogeneous model, MCMC was run for 11,000 iterations, with the first 1000 discarded as burn-in and 10,000 retained for posterior summaries (means and 95 % credible intervals). Posterior predictive distributions of total accrual after 36 and 30 quarters were generated for the HOBIT and BOOST-3 trials, respectively. For the seasonal model, a similar MCMC procedures were implemented, and posterior summaries were generated for each $\lambda_{s}$. Seasonal trends were compared using pairwise posterior probabilities of the form $P\left(\lambda_{S_{\left(j\right)}}>\lambda_{S_{\left(k\right)}}\right)$. The posterior predictive distributions of total accruals for each trial were also obtained.

Model performance was evaluated by using both model fit and predictive accuracy. Model fit was assessed with the Deviance Information Criterion (DIC), where lower values indicate better fit. Predictive performance was evaluated using a rolling internal prediction framework. For each trial, the first k quarters (where k ranges from 1 to J) were treated as observed, and the remaining as unobserved. Posterior samples from each model were then used to generate predictive accruals for the unobserved quarters. The total predicted accrual was calculated by summing the observed and predicted accruals, and the process was repeated 10,000 times to obtain the posterior predictive distribution of total accrual for each value of k under both models. Predictive accuracy was then measured by comparing predicted total accrual with the actual observed accrual using Root Mean Squared Error (RMSE), bias, and standard deviation (SD). For a particular value of observed quarter k, let ${\hat{M}}_{total}^{\left(r\right)}=\sum_{j=1}^{J}m_{j}^{\left(r\right)}$ denote the total predicted accrual from the r-th posterior draw, and $M_{true}=\sum_{j=1}^{J}m_{j}^{true}$ represent the total actual accrual over all J quarters. With R total posterior samples, the performance metrics were computed as:

$$RMSE=\sqrt{\frac{1}{R}\sum_{r=1}^{R}{\left({\hat{M}}_{total}^{\left(r\right)}-M_{true}\right)}^{2}}$$

$$Bias=\frac{1}{R}\sum_{r=1}^{R}\left({\hat{M}}_{total}^{\left(r\right)}-M_{true}\right)$$

$$SD=\frac{1}{R-1}\sum_{r=1}^{R}{\left({\hat{M}}_{total}^{\left(r\right)}-{\bar{M}}_{total}\right)}^{2}\text{,}$$

where ${\bar{M}}_{total}=\frac{1}{R}\sum_{r=1}^{R}{\hat{M}}_{total}^{\left(r\right)}$ is the mean of total predicted accruals from all posterior samples. This process was repeated for increasing numbers of observed quarters (value of k ranging from 1 to 26 for HOBIT, 1 to 21 for BOOST-3), and RMSE, bias, and SD were computed to evaluate model performance over time.

## 4 Results

### 4.1 Application to HOBIT trial

**Fig. 1:**
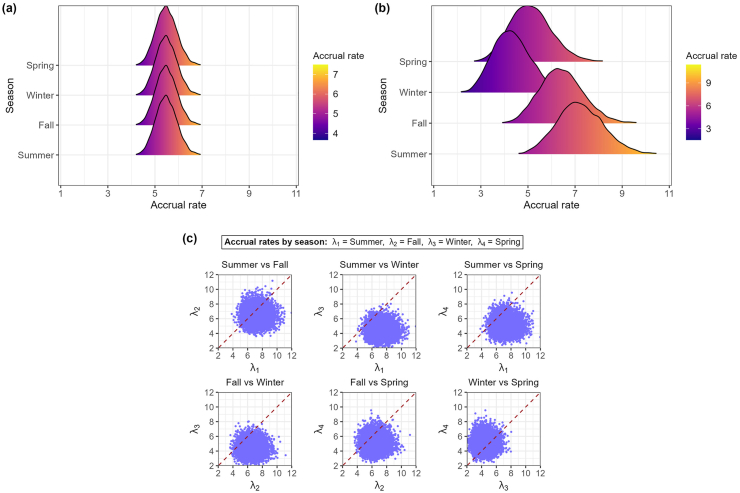
Posterior density estimates of accrual rates by season for the HOBIT trial. Panel (a) shows posterior densities of accrual rates under the homogeneous model. Panel (b) shows posterior densities of accrual rates under the seasonal model. Panel (c) shows pairwise comparisons of seasonal accrual rates $\left(\lambda_{i,}\;\lambda_{j}\right)$ from the seasonal model, where each dot represents a posterior draw for a pair of accrual rates, and the red dashed line denotes the line of equality.

Under the homogeneous model, the posterior mean accrual rate was 5.48 participants per quarter, with a 95 % credible interval of [4.64, 6.39] (Fig. 1(a)). The posterior predictive mean of total accrual after 36 quarters was 193.8 (95 % credible interval: [178, 211]).

When allowing for seasonal heterogeneity using the seasonal model, the highest accrual rate was observed in summer (7.22; 95 % credible interval: [5.41, 9.27]), followed by fall (6.43; 95 % credible interval: [4.76, 8.37]), spring (5.11; 95 % credible interval: [3.51, 6.97]), and winter (4.33; 95 % credible interval: [2.90, 6.11]) (Fig. 1(b); Supplementary Table 1). Compared to the homogeneous model, the seasonal model produced slightly wider credible intervals for accrual rates, reflecting greater uncertainty. Posterior probabilities from pairwise comparisons of seasonal accrual rates also indicated evidence of seasonal variation (Fig. 1(c); Supplementary Table 2). The posterior probability that summer accrual exceeded winter and spring accrual was almost 0.99 and 0.94, respectively, while fall exceeded winter and spring with probabilities of approximately 0.96 and 0.95. Moreover, there was about 70 % probability that summer had the highest accrual and 73 % probability that winter had the lowest. These results suggest a consistent pattern of higher accruals during summer and fall relative to winter and spring. Despite these seasonal differences, the posterior predictive distribution of total accrual after 36 quarters under the seasonal model (mean: 194.6; 95 % credible interval: [178, 213]) was nearly identical to that of the homogeneous model (Supplementary Fig. 1).

**Fig. 2:**
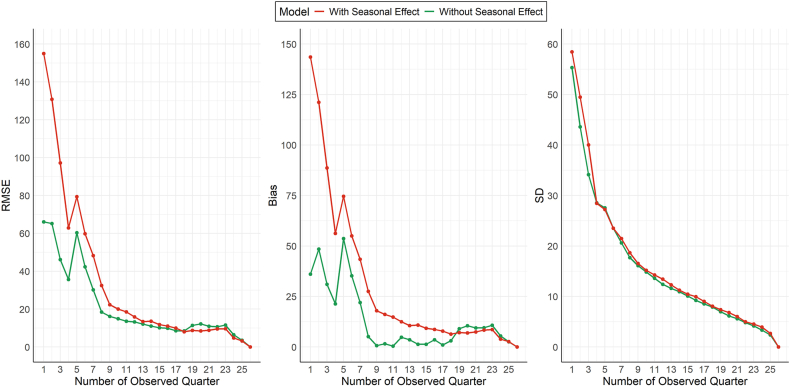
Model performance metrics (Root Mean Squared Error (RMSE), Bias, and Standard Deviation (SD)) over increasing numbers of observed quarters for the HOBIT trial. The red line represents the seasonal model, while the green line represents the homogeneous model.

The model fit assessed by the Deviance Information Criterion (DIC) showed that the seasonal model had a slightly lower DIC value of 124.2, whereas the homogeneous model's DIC was 122.7. This implies that the seasonal model provides a better fit than the homogeneous model, although the difference is minimal. Internal prediction results based on root mean squared error (RMSE), bias, and standard deviation (SD) are shown in Fig. 2. The homogeneous model showed better predictive accuracy during the early and mid-trial periods, as evidenced by consistently lower RMSE, bias, and SD during that time. However, as the number of observed quarters increased, differences between the models diminished, and the seasonal model provided lower RMSE, bias, and SD, specifically with 18 or more observed quarters.

### 4.2 Application to BOOST-3 trial

**Fig. 3:**
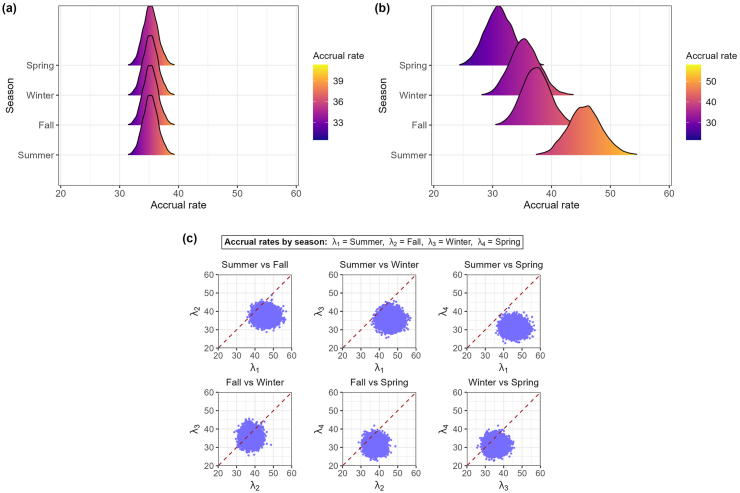
Posterior density estimates of accrual rates by season for the BOOST-3 trial. Panel (a) shows posterior densities of accrual rates under the homogeneous model. Panel (b) shows posterior densities of accrual rates under the seasonal model. Panel (c) shows pairwise comparisons of seasonal accrual rates $\left(\lambda_{i,}\;\lambda_{j}\right)$ from the seasonal model, where each dot represents a posterior draw for a pair of accrual rates, and the red dashed line denotes the line of equality.

The homogeneous model yielded a posterior mean accrual rate of 35.26 participants per quarter, with a 95 % credible interval of [32.82, 37.82] (Fig. 3(a)). The posterior predictive distribution for total accrual after 30 quarters resulted in a mean of 1042 (95 % credible interval: [1001, 1083]).

**Fig. 4:**
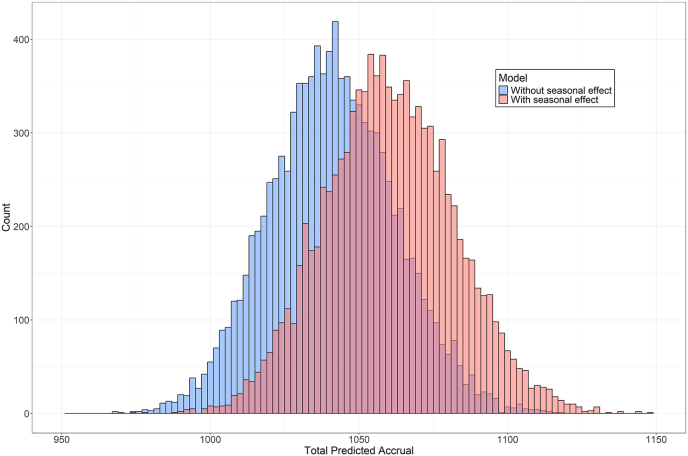
Posterior predictive distribution of total accrual after 30 quarters in the BOOST-3 trial under two models. Blue bars represent predictions from the homogeneous model, and red bars represent predictions from the seasonal model.

The seasonal model provided strong evidence of seasonal effects with heterogeneous posterior accrual rates across seasons (Fig. 3(b); Supplementary Table 3). The highest mean accrual rate was observed in summer (45.67; 95 % credible interval: [40.27, 51.41]), followed by fall (37.41; 95 % credible interval: [32.86, 42.21]), winter (35.46; 95 % credible interval: 30.71, 40.50]), and the lowest in spring (31.10; 95 % credible interval: [26.54, 36.02]). Pairwise comparisons of posterior accrual rates further confirmed these seasonal disparities (Fig. 3(c); Supplementary Table 4). Summer was more likely than any other season to have the highest accrual rate (98.6 %), and spring was most likely to have the lowest (88.2 %). Posterior predictive distributions of total accrual after 30 quarters also reflected seasonal effects (Fig. 4), with the seasonal model showing a rightward shift relative to the homogeneous model, yielding a mean of 1061 participants (95 % credible interval [1019, 1105]).

**Fig. 5:**
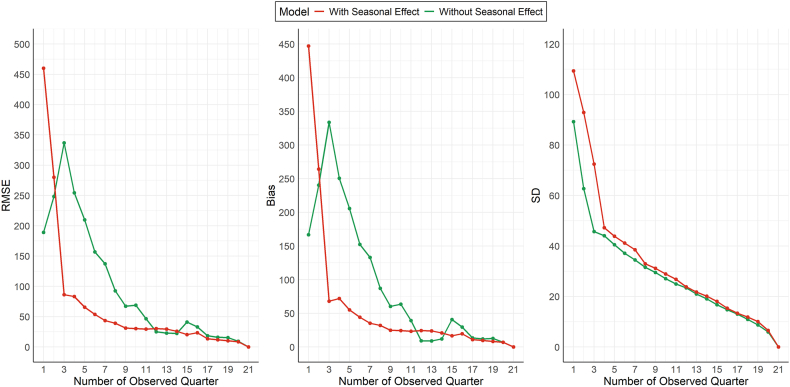
Model performance metrics (Root Mean Squared Error (RMSE), Bias, and Standard Deviation (SD)) over increasing numbers of observed quarters for the BOOST-3 trial. The red line represents the seasonal model, while the green line represents the homogeneous model.

According to the Deviance Information Criterion (DIC), the seasonal model provided a better fit than the homogeneous model in the BOOST-3 trial (DIC: 213.3 vs. 222.6), with a more pronounced difference than in the HOBIT trial. This suggests that accounting for seasonal variation is highly important for accurately modeling accrual patterns in BOOST-3. Internal prediction performance, evaluated using RMSE, bias, and SD across 1 to 21 observed quarters (Fig. 5), showed that the homogeneous model performed slightly better in the first two quarters. However, once multiple seasons were observed, the seasonal model began to capture underlying enrollment patterns more effectively, leading to consistently lower RMSE and bias. Although the seasonal model showed higher variability (SD) in its predictions throughout, the difference narrowed as more quarters were observed, suggesting improved precision over time.

## 5 Discussion

Our study introduces an extension of the homogeneous accrual model that incorporates seasonal effects to improve patient enrollment prediction in clinical trials. Applying both homogeneous and seasonal models in two multicenter trials, HOBIT and BOOST-3, we addressed a critical gap in accrual modeling by demonstrating the importance of incorporating seasonal heterogeneity in patient enrollment. Previous research has shown that enrollment can fluctuate due to weather conditions, holidays, and disease prevalence [[27], [28], [29], [30]]. Our approach responds to the challenge of recruitment inefficiencies, which is a pressing issue in clinical research [[31], [32], [33], [34], [35], [36]], and highlights the importance of incorporating seasonal variation in accrual prediction. In both trials, the homogeneous model performed well in early phases when observed data were limited. However, as more accrual data were accumulated over time, seasonal patterns became apparent, particularly in the BOOST-3 trial. This underscores the limitations of assuming constant accrual across seasons, especially in larger trials.

By allowing accrual rates to vary across seasons, the seasonal model offered greater flexibility in capturing patterns of patient enrollment. In the HOBIT trial, accrual was highest in summer and lowest in winter, yet DIC values and posterior predictive distributions of total accrual were nearly identical under both models. This is likely attributable to the relatively slow accrual rate in HOBIT, which may have limited the impact of seasonal variation. Although pairwise comparison of posterior accrual rates for different seasons indicated seasonal effects, the differences in posterior mean rates across seasons (ranging from 4.33 to 7.22) were not large enough to meaningfully affect predictive performance. As a result, seasonality had only a modest impact in the HOBIT trial.

In contrast, the BOOST-3 trial exhibited much stronger seasonal effects. Accrual rates were considerably higher in this trial, ranging from 31.10 in spring to 45.67 in summer, and the posterior probabilities strongly supported the presence of seasonal heterogeneity. The seasonal model produced a noticeably higher posterior predictive mean for total accrual after 30 quarters and demonstrated a substantially lower DIC value compared to the homogeneous model. Additionally, predictive accuracy assessed by RMSE, bias, and standard deviation, improved more rapidly in the seasonal model for the BOOST-3 trial once multiple seasons were observed. This suggests that accounting for seasonal structure is particularly important in trials with higher accrual rates and longer durations.

Overall, our findings suggest that the utility of incorporating seasonality into accrual modeling is context-dependent. For trials with lower accrual rates, such as HOBIT, the homogeneous model may be sufficient for accrual prediction. However, in larger trials with higher accrual rates like BOOST-3, the seasonal model offers greater advantages in both model fit and prediction accuracy. This can be particularly useful for adaptive clinical trials, where the seasonal model can be used at any given interim time point to predict the accrual at the next interim look while accounting for seasonal variation. This allows study coordinators to monitor accrual patterns and, if necessary, adjust season-specific recruitment strategies or reallocate resources to ensure that accrual is maintained as planned at interim time points and that the target accrual is achieved by study completion. We also note that the seasonal model is fully compatible with alternative time units. For trials where there is appropriate clinical justification, our model can be readily adapted by redefining the time units from quarterly to weekly or monthly and making corresponding adjustments to the gamma priors. Future work should evaluate the generalizability of our modeling approach across diverse trial designs and disease areas. As both HOBIT and BOOST-3 focused on severe traumatic brain injury, further validation of the seasonal model is recommended, along with potential extensions as needed. Moreover, integrating seasonality into adaptive trial monitoring frameworks may also be explored to enhance decision-making during ongoing enrollment.

## 6 Conclusion

This study demonstrates the necessity of incorporating seasonal effects into Bayesian accrual modeling for the prediction of patient enrollment, which is critical for clinical trial planning, monitoring, resource allocation, and adaptive design implementation. While the homogeneous model offers simplicity and performs well in early accrual periods, the seasonal model provides a better fit and more accurate accrual prediction when enrollment patterns vary over different seasons. Our application to the HOBIT and BOOST-3 trials illustrates that accounting for seasonal heterogeneity can enhance prediction accuracy, particularly in large, long-duration trials with high accrual rates.

## CRediT authorship contribution statement

**Mohammod Mahmudur Rahman:** Writing – review & editing, Writing – original draft, Visualization, Software, Methodology, Formal analysis, Data curation. **Md Saiful Islam Saif:** Writing – review & editing, Writing – original draft, Methodology, Formal analysis. **Jonathan Beall:** Writing – review & editing, Supervision. **Renee’ L. Martin:** Writing – review & editing. **Gaylan L. Rockswold:** Writing – review & editing, Funding acquisition. **William G. Barsan:** Writing – review & editing, Funding acquisition. **Frederick K. Korley:** Writing – review & editing, Funding acquisition. **Robert Silbergleit:** Writing – review & editing. **Valerie Stevenson:** Writing – review & editing. **Byron Gajewski:** Writing – review & editing, Supervision, Project administration, Methodology, Funding acquisition, Conceptualization.

## Funding

National Institute of Neurological Disorders and Stroke of the National Institutes of Health, Grant/Award Numbers: U01NS095926 and U01NS099046.

## Declaration of competing interest

The authors declare the following financial interests/personal relationships which may be considered as potential competing interests: Byron Gajewski, William G Barsan, Gaylan L Rockswold, Frederick K Korley reports financial support was provided by National Institute of Neurological Disorders and Stroke of the National Institutes of Health. If there are other authors, they declare that they have no known competing financial interests or personal relationships that could have appeared to influence the work reported in this paper.

## Data Availability

Data will be made available on request.
