# Supplementary material for: A Bayesian model with seasonal effects for predicting accrual in clinical trials: Application to HOBIT and BOOST-3 trials for severe traumatic brain injury
Source: Contemp Clin Trials Commun. 2025 Dec 5;48:101586. doi: 10.1016/j.conctc.2025.101586 (PMC12723122; doi:10.1016/j.conctc.2025.101586)
Supplement: Multimedia component 1 [file mmc1.docx]

**Supplementary Files**


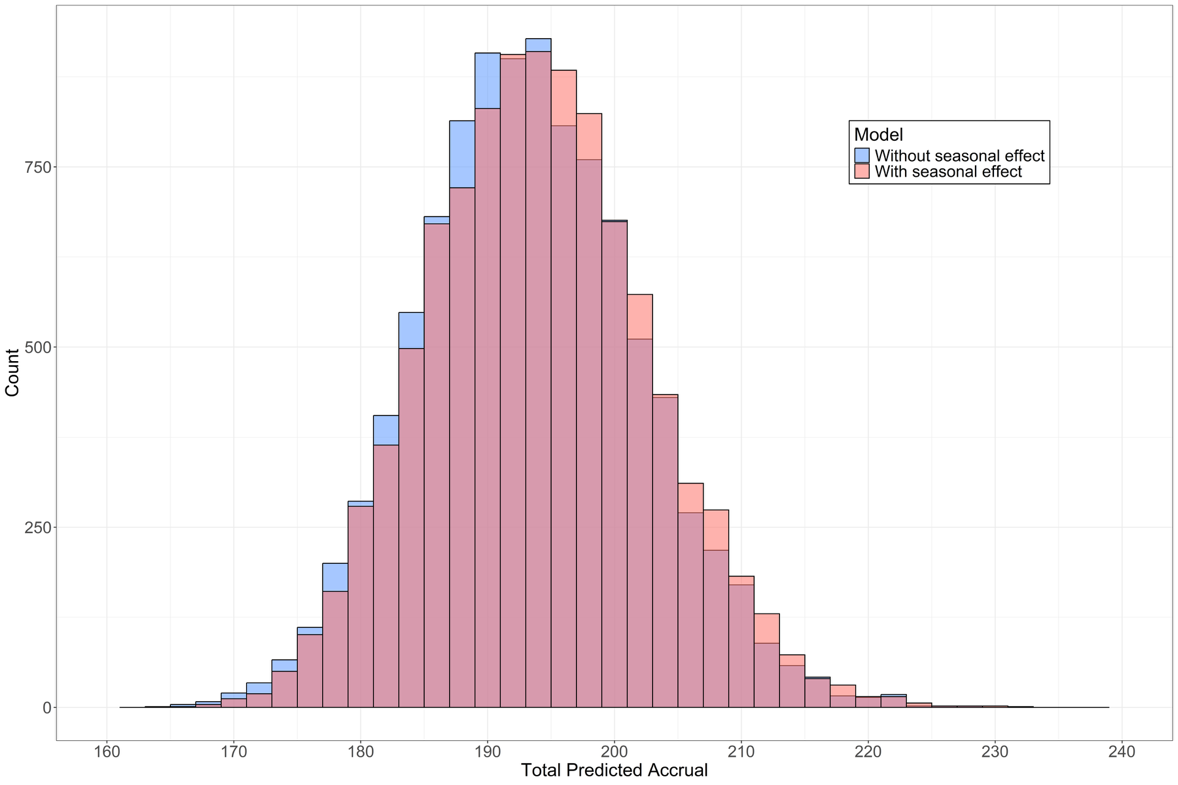


**Supplementary Figure 1:** Posterior predictive distribution of total accrual after 36 quarters in the HOBIT trial under two models. Blue bars represent predictions from the homogeneous model, and red bars represent predictions from the seasonal model.

**Supplementary Table 1:** Posterior summary of accrual rates by season under the seasonal model for the HOBIT trial.

| **Summary** | $\boldsymbol{\lambda}_{\boldsymbol{1}}$ **(Summer)** | $\boldsymbol{\lambda}_{\boldsymbol{2}}$ **(Fall)** | $\boldsymbol{\lambda}_{\boldsymbol{3}}$ **(Winter)** | $\boldsymbol{\lambda}_{\boldsymbol{4}}$ **(Spring)** |
| --- | --- | --- | --- | --- |
| **Mean** | 7.22 | 6.43 | 4.33 | 5.11 |
| **Minimum** | 3.83 | 3.62 | 1.91 | 2.42 |
| **Maximum** | 11.97 | 11.17 | 8.03 | 9.55 |
| **95% Credible Interval** | (5.41, 9.27) | (4.76, 8.37) | (2.90, 6.11) | (3.51, 6.97) |

**Supplementary Table 2:** Posterior probabilities of pairwise comparisons of seasonal accrual rates under the seasonal model for the HOBIT trial.

| **Comparison** | **Probability** |
| --- | --- |
| $\lambda_{1}> \lambda_{2}$ | 72.02 % |
| $\lambda_{1}> \lambda_{3}$ | 98.81 % |
| $\lambda_{1}> \lambda_{4}$ | 94.08 % |
| $\lambda_{2}> \lambda_{3}$ | 95.57 % |
| $\lambda_{2}> \lambda_{4}$ | 84.79 % |
| $\lambda_{4}> \lambda_{3}$ | 74.18 % |
| $\lambda_{1}=\max({\lambda_{1},\lambda}_{2},\lambda_{3},\lambda_{4})$ | 69.86% |
| $\lambda_{3}=\min({\lambda_{1},\lambda}_{2},\lambda_{3},\lambda_{4})$ | 72.86% |

**Supplementary Table 3:** Posterior summary of accrual rates by season under the seasonal model for the BOOST-3 trial.

| **Summary** | $\boldsymbol{\lambda}_{\boldsymbol{1}}$ **(Summer)** | $\boldsymbol{\lambda}_{\boldsymbol{2}}$ **(Fall)** | $\boldsymbol{\lambda}_{\boldsymbol{3}}$ **(Winter)** | $\boldsymbol{\lambda}_{\boldsymbol{4}}$ **(Spring)** |
| --- | --- | --- | --- | --- |
| **Mean** | 45.67 | 37.41 | 35.46 | 31.10 |
| **Minimum** | 35.83 | 28.73 | 25.59 | 22.68 |
| **Maximum** | 56.94 | 48.74 | 45.68 | 41.88 |
| **95% Credible Interval** | (40.27, 51.41) | (32.86, 42.21) | (30.71, 40.50) | (26.54, 36.02) |

**Supplementary Table 4:** Posterior probabilities of pairwise comparisons of seasonal accrual rates under the seasonal model for the BOOST-3 trial.

| **Comparison** | **Probability** |
| --- | --- |
| $\lambda_{1}> \lambda_{2}$ | 98.80 % |
| $\lambda_{1}> \lambda_{3}$ | 99.74 % |
| $\lambda_{1}> \lambda_{4}$ | 100 % |
| $\lambda_{2}> \lambda_{3}$ | 71.66 % |
| $\lambda_{2}> \lambda_{4}$ | 96.94 % |
| $\lambda_{3}> \lambda_{4}$ | 89.68 % |
| $\lambda_{1}=\max({\lambda_{1},\lambda}_{2},\lambda_{3},\lambda_{4})$ | 98.58 % |
| $\lambda_{4}=\min({\lambda_{1},\lambda}_{2},\lambda_{3},\lambda_{4})$ | 88.23 % |
